# A psychosocial intervention for the management of functional dysphonia: complex intervention development and pilot randomised trial

**DOI:** 10.1186/s40814-018-0240-5

**Published:** 2018-02-08

**Authors:** Vincent Deary, Elaine McColl, Paul Carding, Tracy Miller, Janet Wilson

**Affiliations:** 1 Department of Psychology, Faculty of Health and Life Sciences, Northumbria University, Newcastle, NE1 8ST UK; 2 Institute of Health and Society, Newcastle University, Richardson Road, Newcastle, NE2 4AX UK; 3 School of Allied Health I, Faculty of Health Sciences, Australian National Catholic University, KB02, Brisbane, Queensland 4014 Australia; 4 Department of Otolaryngology and Head and Neck Surgery, Freeman Hospital, Newcastle University, Newcastle, NE7 7DN UK

**Keywords:** Medically unexplained symptoms, Cognitive behavioural therapy, Speech and language therapy, Functional dysphonia, Pilot randomised controlled trial

## Abstract

**Background:**

Medically unexplained loss or alteration of voice—functional dysphonia—is the commonest presentation to speech and language therapists (SLTs). Besides the impact on personal and work life, functional dysphonia is also associated with increased levels of anxiety and depression and poor general health. Voice therapy delivered by SLTs improves voice but not these associated symptoms. The aims of this research were the systematic development of a complex intervention to improve the treatment of functional dysphonia, and the trialling of this intervention for feasibility and acceptability to SLTs and patients in a randomised pilot study

**Methods:**

A theoretical model of medically unexplained symptoms (MUS) was elaborated through literature review and synthesis. This was initially applied as an assessment format in a series of patient interviews. Data from this stage and a small consecutive cohort study were used to design and refine a brief cognitive behavioural therapy (CBT) training intervention for a SLT. This was then implemented in an external pilot patient randomised trial where one SLT delivered standard voice therapy or voice therapy plus CBT to 74 patients. The primary outcomes were of the acceptability of the intervention to patients and the SLT, and the feasibility of changing the SLT’s clinical practice through a brief training. This was measured through monitoring treatment flow and through structured analysis of the content of intervention for treatment fidelity and inter-treatment contamination.

**Results:**

As measured by treatment flow, the intervention was as acceptable as standard voice therapy to patients. Analysis of treatment content showed that the SLT was able to conduct a complex CBT formulation and deliver novel treatment strategies for fatigue, sleep, anxiety and depression in the majority of patients. On pre-post measures of voice and quality of life, patients in both treatment arms improved.

**Conclusion:**

These interventions were acceptable to patients. Emotional and psychosocial issues presented routinely in the study patient group and CBT techniques were used, deliberately and inadvertently, in both treatment arms. This CBT “contamination” of the voice therapy only arm reflects the chief limitation of the study: one therapist delivered both treatments.

**Trial registration:**

Registered with the ISRCTN under the title: *Training a Speech and Language Therapist in Cognitive Behavioural Therapy to treat Functional Dysphonia - A Randomised Controlled Trial*.

Trial Identifier: ISRCTN20582523 Registered 19/05/2010; retrospectively registered. http://www.isrctn.com/ISRCTN20582523

## Background

Whilst the classification and nomenclature of voice disorders remain disputed [1], “functional dysphonia” can be used (and is used here) to denote an alteration or loss of voice in the absence of an organic disorder, or where the observed pathology is insufficient to explain the vocal symptoms. Thus defined, functional dysphonia is the commonest disorder presenting to UK voice clinicians, accounting for up to 40,000 new cases per year [2]. It is known to affect communication in all contexts and is related to impaired personal and work relationships, low self-esteem and reduced quality of life [3]. In addition, people with functional dysphonia also suffer from increased levels of anxiety, depression and poor general health [4, 5]. Voice therapy, delivered by speech and language therapists, has been shown to improve voice quality in functional dysphonia patients, but there is no evidence to date of any effect on their more general well-being [6]. The aim of our research programme was to develop and pilot a new psychosocial intervention aimed at improving both voice and well-being in this patient group.

As defined above, functional dysphonia can be classified as a medically unexplained symptom (MUS) [1]. MUS in the absence of physical pathology form a considerable health care burden, with around 50% of referrals in specialist clinics being in some way medically unexplained [7]. For many medically unexplained conditions, there is evidence for the effectiveness of cognitive behavioural therapy (CBT), a multi-component complex intervention involving a mixture of changing behaviours, such as symptom lead patterns of activity avoidance, and changing beliefs, such as catastrophic interpretation of symptoms [8]. In attempting to improve both the treatment and understanding of functional dysphonia, it seems appropriate to employ theoretical and clinical insights derived from previous applications of a CBT model in other MUS to this speech disorder.

The application of the CBT model to these other conditions has been successful to the extent that many MUS have been to some degree explained, and improved, as has their co-morbid distress [9]. Initially, the therapy was typically delivered by highly specialised professionals trained in psychiatry, clinical psychology or cognitive behavioural therapy. Recently, with a view to making this type of therapy more widely available in a cost-effective manner, there has been a move to training non-specialists such as nurses and allied health professionals to deliver CBT. For example, there have been attempts to improve patient outcomes in diabetes and irritable bowel syndrome through training practice nurses in CBT [10, 11].

In a review of the nature and treatment of functional dysphonia [12], Baker states that the training of speech and language therapists to assess and treat the psychosocial issues associated with functional dysphonia is a “ethical and professional obligation” ([12] page 103). We therefore aimed to develop, in several stages, a CBT intervention for delivery by speech and language therapists (thus requiring professional behaviour change) and then to test its feasibility and acceptability in an external pilot randomised controlled trial, comparing the speech and language therapist (SLT)-delivered CBT to usual care.

**Fig. 1:**
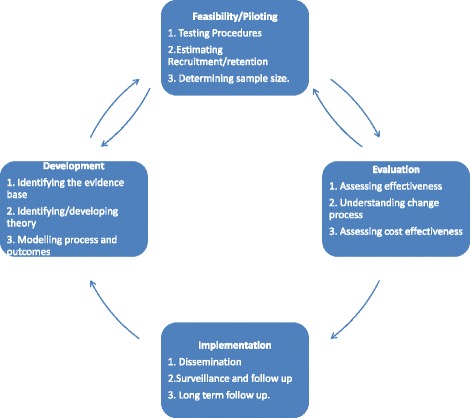
The Medical research council framework for complex intervention development

A behaviour change intervention delivering CBT techniques to a patient presenting with a physical problem constitutes a “complex intervention”; it is complex both by virtue of the multi-component nature of the intervention and by the intended mode of delivery—the training of a health professional. As several authors have noted [13–15], there is surprisingly little evidence or consensus around the development and evaluation of complex interventions, particularly regarding the earlier developmental stages. To address this, the Medical Research Council (MRC) published guidelines [15] in a model which acknowledges the iterative, cyclical nature of the process (see Fig. 1).

In the development phase, the theoretical and empirical grounds of the intervention need to be established. Typically, this involves a review of the evidence base and an identification or development of the underpinning theory for the intervention. In the field of behaviour change research, there is increasing emphasis on the importance of basing interventions on theory [13, 16]. There is however less consensus, and little evidence, for what should happen in the modelling phase, where the nature of the behaviour change intervention is specified. Suggested methods include evidence review, patient interviews (group and individual) and expert consultations [13, 14, 17]. The present paper describes a systematic, iterative process of intervention development which is theoretically based, patient centred and guided by the MRC framework from modelling through to piloting.

## Methods

### Stage 1: development

#### Theory selection.

The first step in the identification of a theory and a theoretical model was a comprehensive review of the literature of explanatory processes and models of MUS in general [9]. This process identified a general explanatory model of functional symptoms as having predisposing, precipitating and perpetuating factors. The perpetuating factors included physical, behavioural, cognitive, affective and social components. This expanded CBT model of MUS [9] formed the theoretical basis of the current study of FD patients.

#### Model building

Next, the literature on predisposing, precipitating and perpetuating factors in functional dysphonia was reviewed in the context of the expanded CBT model of MUS. Evidence was found for predisposing vulnerabilities (gender, personality, occupation, other MUS); precipitating triggers (life events, viruses) and perpetuating factors (dysregulation of the laryngeal and paralaryngeal muscles, anxiety and depression) (for full review see [1].

#### Model development—patient interviews

Eight patients with functional dysphonia were interviewed by one member of the team (VD, a cognitive behavioural therapist) using an assessment structure based on the expanded CBT model identified above. This was used to elicit as wide a range of predisposing, precipitating and perpetuating factors of patients’ problems as possible. Individualised formulations, describing the interaction of multiple factors in the onset and maintenance of functional dysphonia and its associated distress, were worked out in session in collaboration with patients.

#### Initial development and delivery of the intervention.

Common factors, themes and patterns of interaction identified from the patient interviews and formulations as being important in the causation and maintenance of functional dysphonia were used alongside the theory and modelling data to develop an individually adaptable, expanded CBT model of functional dysphonia. This formed the basis of the speech and language therapist training package. This training was initially piloted in a small consecutive cohort study [18].

#### Refinement of the functional dysphonia model

Two notable further insights were gained through clinical supervision during this small cohort study and through discussion with speech and language therapists. Functional dysphonia patients routinely reported exhaustion, with disturbed patterns of activity, rest and sleep. They also tended to report perfectionist tendencies. This leads to a questionnaire-based case-control study into these aspects of dysphonia, which confirmed that this group was significantly more fatigued and perfectionist than matched normal controls [19]. This further shaped the CBT model and training.

#### Assessing and maximising the likelihood of intervention uptake

The prior consecutive cohort study [18] provided evidence that, for at least one speech and language therapist, delivering the CBT was feasible and acceptable. To further tailor the training for the generic and specific context in which it was to be implemented, VD carried out interviews with individual therapists, clinical teams and a voice special interest group. This process gave an understanding of how presenting problems other than voice issues (particularly anxiety, depression and personality problems) were routinely dealt with by SLTs in their management of functional dysphonia patients. Next, the SLT to be trained to deliver the CBT intervention (TM) was interviewed, to establish training needs, learning style and normal practice in the management of functional dysphonia and to establish a protocol for how emotional issues were to be dealt with in the usual care randomised controlled trial (RCT) arm.

### Stage 2: feasibility—an external pilot randomised controlled trial

As a preliminary, a further set of patient interviews were conducted with greater attention to factors highlighted in the initial intervention development stages. These interviews confirmed the findings on perfectionism, fatigue, disordered activity, rest and sleep. Furthermore, the interviews supported the feasibility of addressing these with patients through developing a shared multi-factorial understanding of their condition. The insights gained from the above stages were used to further refine the model, the training of the therapist and the design and conduct of the pilot RCT. The objectives of this trial were as follows:

#### Acceptability and feasibility objectives

- To assess the feasibility and acceptability of procedures and methods for trial participant identification, recruitment and data collection.
- To assess the feasibility and acceptability of the CBT intervention to a SLT by evaluating their ability to integrate CBT training into clinical practice and by evaluating the amount and nature of supervision required to embed the CB intervention in usual care.
- To assess the fidelity of delivery, acceptability and clinical utility of a CBT intervention to functional dysphonia patients.
- To test the sensitivity to change of a selection of candidate outcome measures

These objectives were measured and monitored through recruitment and retention rates (patient acceptability of intervention and trial procedures), through observation of the training process (therapist acceptability) and through monitoring the process and content of the CBT participants’ treatment, in clinical supervision, case recordings, case notes and case summaries (fidelity, therapist and patient acceptability and feasibility, clinical utility). The main outcomes were the feasibility and acceptability estimates, assessed as described above. In addition, measures were taken of voice (Voice Performance Questionnaire, Carding et al. 1999), general health (General Health Questionnaire, Goldberg and Williams 1988) and psychological distress (Hospital Anxiety and Depression Scale Zigmond and Snaith, 1983, at baseline, at discharge from treatment (usually 6–8 sessions happening every other week) and 6 months after the end of treatment. These outcomes were assessed with regard to their acceptability and responsiveness to change, to assist in powering a future trial and will be the subject of a separate paper.

#### Design

The trial was a single-centre external pilot, patient randomised controlled trial with two arms: standard voice therapy versus voice therapy plus CBT, both delivered by a single SLT (TM), experienced in treating functional dysphonia.

The study was conducted at the Speech Voice and Swallowing Clinic of the Freeman Hospital, Newcastle upon Tyne, UK, between October 2007 and August 2010. Participants who remained in treatment or follow-up after this point continued to receive fully supervised treatment. Ethical permission was sought and obtained from Newcastle and North Tyneside Research Ethics Committee 1 (ethics reference number: 07/H0906/118). The trial was registered with the ISRCTN under the title: *Training a Speech and Language Therapist in Cognitive Behavioural Therapy to treat Functional Dysphonia - A Randomised Controlled Trial*. Trial Identifier: ISRCTN20582523.

#### Inclusion/exclusion criteria

Study participants were patients who had been referred to the Speech and Voice Clinic, for assessment of their dysphonia. Patients were screened by endoscopy, which excluded the presence of an injury, a lesion or a movement disorder in the patient’s voice box. Patients who had been thus diagnosed as having functional dysphonia were approached regarding entry into the trial. They were given participant information sheets describing the study in detail and at least 24 h to consider participation.

For the sake of generalisability, inclusion criteria were as broad as possible. Patients were considered eligible for randomisation if they were aged 18 or over and presented with an alteration or loss of voice where there was no evidence of a non-functional reason for vocal impairment (other than vocal nodules), a score of ≥ 1 on the overall Grade component of the clinician-rated voice quality Grade Roughness Breathiness Asthenia Strain Scale (GRBAS) [20] and a score of ≥ 20 on the self-rated Vocal Performance Questionnaire (VPQ) [21] (a self-report measure of voice quality and voice related disability rated 12–60 with 12 being normal). Patients were excluded from the trial if they had any of the following: previous experience of CBT for their voice problem; an acute or ongoing serious medical illness or severe mental health problem which was likely to interfere with their ability to comprehend, engage and/or comply with treatment; a learning disability; and a mild vocal condition which did not merit a full course of treatment.

#### Interventions—usual care: voice therapy

The control condition of “usual care” aimed to be as close to standard voice therapy practice as possible. Patients were offered an average of six to eight sessions every 2 weeks of approximately 1 h of voice therapy, although length and number of sessions were allowed to vary as needed. The content typically had the following elements: vocal hygiene and education (such as maintaining adequate vocal hydration); elimination of voice misuse and abuse (such as excessive throat clearing or shouting); breath control and coordination with phonation; and in-session and between-session exercises to promote vocal flexibility and resonance. When emotional issues arose in the course of therapy sessions, the SLT employed non-directive counselling skills, whereby patients were encouraged to speak about difficult issues, reflecting TM’s normal pre-CBT practice.

#### Interventions—the CBT intervention

In the experimental CBT arm, in addition to the standard voice therapy, patients also received the following CBT elements. As with usual care, treatment sessions lasted approximately 1 h.

#### Assessment and formulation

The CBT assessment identified the predisposing, precipitating and perpetuating factors. It was derived from the CBT model of functional dysphonia previously described. This information formed the basis of a formulation that attempted to explain how current factors might be interacting to maintain both poor voice and general distress, and how these had developed through the interaction of predisposing and precipitating factors. This formulation formed the basis for both treatment delivery (by TM) and treatment supervision (by VD). The ability to reach an agreed formulation with the patient was also a key measure of the acceptability of CBT for the patient group.

#### Treatment techniques

As each patient had an individualised formulation, no two treatments were identical, but they typically consisted of a mixture of the following treatment techniques. For low energy and low mood, patients were advised gradually to do more, in a structured planned way, and gradually to resume activities that used to be done for enjoyment and achievement. These evidence-based methods [22] are relatively simple and hence easily transmissible from trainer to therapist and from therapist to patient. Graded exposure was used to address anxiety-based issues [23]. People who are anxious tend to avoid what they are anxious of (and thus become more anxious) encouraging people to gradually confront difficult situations in a planned and structured way and at their own pace is the best evidence-based treatment [23]. This work also incorporated simple cognitive techniques, such as helping the patient identify what kind of anxious thoughts they might be having about avoided situations and helping them to test out the reality of these thoughts by confronting the situation in a safe, planned manner. Cognitive work was thus conceptualised as being an adjunct and aide to behavioural change. In addition, the therapist was trained in specific cognitive techniques for the negative aspects of perfectionism such as very high self-standards and self-criticism [24]. Other common unhelpful beliefs concerned the best way to manage voice and other physical symptoms, with patients often interpreting symptoms as harmful and as a cue to stop activity and to socially withdraw, thus keeping going a cycle of physical dis-use, low energy and low mood. Cognitive techniques, such as guided questioning during therapy sessions and thought diaries in between sessions, helped patients to identify their unhelpful beliefs and test them out by looking for evidence both for and against them.

These assessment and treatment techniques were taught to TM over a total of 7 days, over a 2-week period, by VD, with extensive use of skills rehearsal and supported by a full training manual. Patient implementation was supported with a patients’ manual, outlining each of the above approaches, with examples.

#### Monitoring and supervision

All treatment sessions were recorded using digital audio equipment. All patients in the CBT arm were reviewed weekly using a CBT supervision framework where the individualised formulation formed the basis for case discussions between TM and VD. TM’s management of those randomised to usual care (voice therapy alone) was supervised as usual by PC (another experienced speech and language therapist). To monitor for the intrusion of CBT techniques into usual care, TM identified patients in this cohort where distress was an issue and VD listened to recorded sessions and asked her to describe her treatment approach. Detailed clinical notes were also kept by TM of session content for both arms of the trial. These provided content for supervision and allowed the performance of fidelity, feasibility and acceptability analyses of the intervention for both participants and therapist.

#### Sample size

As this was an external pilot RCT, the primary determinant of the sample size was pragmatic, i.e. the throughput of the unit over the period of the trial. No formal power calculation was used but the guideline of Lancaster et al. [25] was followed which suggests that in an external pilot trial, 30 patients per arm may be sufficient to estimate the parameters of interest for a larger trial. Factoring in estimates of likely non-suitability, we anticipated being able to recruit this number over 18 months. We continued to recruit up to this latter time-point, resulting in 74 patients randomised to the study.

#### Randomisation, allocation concealment and blinding

Once a patient consented, they were randomly allocated either to voice therapy alone or voice therapy plus CBT. An independent researcher at Newcastle Clinical Trials Unit prepared a randomisation list, using permuted blocks of random sizes to reduce risk of breach of concealment of allocation. Sequentially numbered, opaque envelopes were prepared and provided to the clinic. The randomisation envelope was opened by the speech and language therapist in the presence of the patient following receipt of written consent. Patient details, date and time of randomisation were recorded, to maintain an audit trail of randomisation. Neither participant nor the therapist (TM), who was also responsible for data collection, nor the researcher responsible for data analysis (VD) was blind to treatment allocation.

#### Data analysis and use

Data were analysed in the following stages. Rates of recruitment, retention and protocol violations were analysed with respect to the feasibility and acceptability of the interventions from patient and therapist standpoints. Success of randomisation was assessed by informally comparing both groups’ baseline characteristics. A tally of problem formulations, treatment targets and treatment techniques was done in the CBT group to assess the feasibility of transmitting CBT techniques to an SLT, and the acceptability of CBT techniques to patients. A tally was also done of the content of any voice therapy only sessions where emotional issues formed a significant part of the work.

Full trial protocol is available [26].

## Results

The following is reported to the extended CONSORT guidelines for reporting pilot and feasibility studies [27].

### Feasibility and acceptability

We judged the feasibility and acceptability of the intervention according to the following criteria: comparable rates of drop-out between both trial arms (patient acceptability); evidence of a CBT formulation being possible for the majority of clients in the CBT arm (clinician and patient acceptability); evidence of CBT treatment strategies for the majority of clients in the CBT trial arm (clinician and patient acceptability). Evidence of sensitivity to change in candidate outcome measures (questionnaire acceptability).

#### Treatment flow

**Fig. 2:**
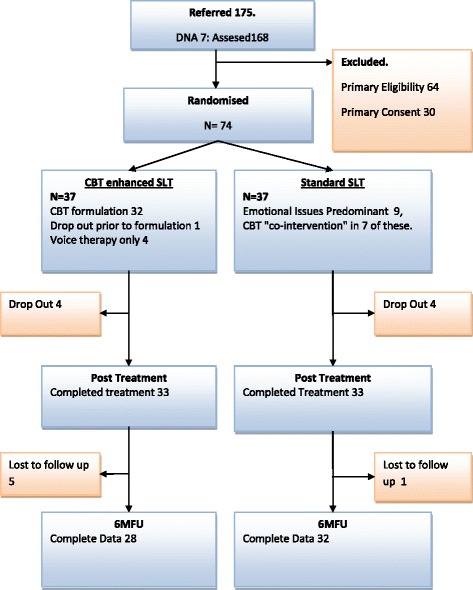
Treatment flow

One hundred seventy-five patients were referred by GPs or consultants to the Speech and Voice Clinic of the Freeman Hospital, over the recruitment period of the trial (October 2007–September 2009). Of the 175 that were referred, 7 did not attend their appointments so 168 (96% of referrals) were assessed for eligibility. Of these, 64 (38% of those screened) did not fulfil the inclusion criteria: 46 were only mildly dysphonic, i.e. their GRBAS Grade item score was less than 1 and/or their VPQ score was less than 20. A further ten had other voice disorders: six were unspecified, two had spasmodic dysphonia, one had puberphonia and one had a vocal cord cyst. Three had a serious medical complaint and three had a serious mental health issue. One had insufficient English and one was under 18 years old. The rate of eligibility amongst those assessed was therefore 62% (104/168). Of those who were apparently eligible, a further 30 (29%; 30/104) did not consent to be randomised. Of these, two were in psychotherapy and four had had recent psychotherapy; two did not like the idea of psychotherapy; one could not commit to the time involved in therapy; one had too far to travel and one had an on-going complaint against the NHS Trust. Nineteen did not give a reason. The remaining 74 patients (71% of those eligible) were recruited to the trial; 37 were randomised to each treatment group. These were the figures originally intended (see Fig. 2).

Considering drop outs from treatment as a measure of treatment acceptability/feasibility, there were 4/37 (10.8%) in the CBT group and 4/37(10.8%) in the SLT group. At the 6-month follow-up, a further five patients did not attend the appointment or return questionnaires in the CBT group; an additional one patient did not attend appointment or return questionnaires in the usual care group. Thus follow-up attrition was 24.3% for CBT and 13.5% for usual care. Reasons for this will be considered in the “Discussion”.

#### Protocol adherence and violations

Both protocol adherence and violations were monitored on an on-going basis through clinical supervision of the SLT’s case load, audio recordings of sessions and case notes.

An individualised CBT formulation was possible with, and acceptable to, 32 out of 37 participants (86.5%). Of the five where formulation was not possible, three reported dysphonia as their sole problem with general mental and physical health being otherwise good. These three participants received voice therapy only. The fourth participant reported a co-morbid anger management problem, already being managed by medication, and also received voice therapy only. Thus, four participants in the CBT arm received voice therapy only. One further patient allocated to the CBT group dropped out early in treatment before the feasibility of a formulation could be determined. There were three other notable cases in the CBT group. One participant was severely depressed and required joint sessions delivered by the SLT (TM) with the CBT supervisor (VD), in liaison with their general practitioner. One participant contracted tuberculosis and required a long mid treatment break. One participant, whose anxiety disorder had been responding well to CBT, suffered a relapse following a violent trauma in her family and needed an extended treatment course over the following 18 months.

In the SLT group, three dropped out after the first session. Nine participants (24%; 9/37) had emotional issues which formed a substantial part of the treatment plan; for seven (18.9%), there was either notable CBT influence in SLT treatment sessions or the patient received other psychological intervention (by another health professional) external to the pilot trial. This “contamination” or co-intervention will be discussed further below.

#### Baseline data

Table 1 shows the baseline characteristics of the groups and also the mean number of sessions in each of the treatment arms. Groups were well matched on most characteristics at baseline, demonstrating the success of randomisation. Demographically, there were twice as many men in the SLT group than in the CBT group, but proportions were small in both groups (16.2 and 8.1% respectively). There were more professional voice users, e.g. teachers, call centre workers and counsellors, in the SLT (62.2%) group than in the CBT group (37.9%). Some measures were missing at baseline, thus *N* varies in the table.

**Table 1:** Baseline characteristics of the two arms of the pilot RCT

	*N*	SLT only	Caseness *N*(%)	*N*	CBT	Caseness *N*(%)
Age	37	45.8(13.5)		37	43.5 (16.3)	
Male		6 (16.2%)			3(8.1%)	
Female		31(83.8%)			34(91.9%)	
Voice user		23(62.2%)			14(37.9%)	
Measures		Mean(sd)			Mean(sd)	
GRBAS	37	1.6(0.7)		37	1.8(0.8)	
HAD Depression	31	5.3(4)	12(39%)	34	6.5 (4.2)	11(32%)
VPQ	37	31 (7.9)		36	33.4 (8.9)	
HAD Anxiety	31	8.4 (4.7)	21(68%)	34	8.9(4.4)	19(56%)
GHQ-28	32	29.4(13.6)	21(66%)	34	28.1(14.3)	21(62%)
Sessions		6.2(4.1)			7.5(4.1)	

On all baseline outcome scores, patients in both arms were comparable. The mean General Health Questionnaire-28 scores [28] of participants in each arm exceeded the caseness threshold; 56.8% of participants in each arm had General Health Questionnaire (GHQ)-28 scores equalling or exceeding the caseness threshold of 22. On Hospital Anxiety And Depression Questionnaire (HAD) [29] measures of anxiety and depression, where the caseness cut-off is 8 for both factors, the mean of both groups was clinically significant on anxiety only. The percentages clinically anxious were 56.8 and 51.3% in the SLT and CBT arms respectively. Mean HAD depression scores fell below the caseness threshold with 32.4% being cases in the SLT arm and 29.7% in the CBT arm. Mean fatigue levels were higher than the general population norm (13.72, 95% CI 13.65–13.79) [30] in both groups. The numbers of sessions of therapy received were comparable. In terms of impact of baseline scores, although numbers were too small to establish significance, there was an interesting trend. Participants with higher scores in vocal disability tended to drop out of the SLT arm, and participants with lower measures of anxiety, depression and fatigue tended to drop out of the CBT arm.

#### CBT treatment content and fidelity as measures of treatment acceptability and feasibility

Fidelity of delivery, feasibility and acceptability to patients of the CBT intervention was assessed by monitoring the content of the sessions via real-time clinical supervision and retrospective content analysis of TM’s contemporaneous case notes. As mentioned above, CBT case formulation was feasible in 32 out of the 37 cases (86%) with the exceptions being mostly “pure” voice disorders. The acceptability and feasibility for participants (and by implication also for the SLT) were further analysed by assessing in what proportion of participants it was possible to identify the main hypothesised targets for CBT treatment, and in what proportion of participants CBT techniques were attempted.

Fatigue was identified as a clinical issue in 27 (72.9%) of the participants in the CBT arm. Of these, 20 (74.1%) attempted graded activity interventions, the main evidence-based intervention for fatigue. Of the seven who did not, four were treatment drop outs and two chose only to engage with the voice therapy aspects of treatment. One participant did discuss the general principles of graded activity but, unlike the others, did not keep diaries or set targets. This more discursive approach was classified as generic counselling in our feasibility analysis, being closer in content to the SLT’s pre-existing clinical skills. Sleep problems, mainly in sleep onset and/or maintenance, were a clinical issue for 25 participants (67.6%). Of these, 18 (72%) attempted sleep management strategies. As with fatigue, of the seven who did not attempt sleep management strategies, four were treatment drop outs, two engaged only in voice therapy and one discussed principles without formally applying them.

Low mood was identified as a clinical issue for 23 (62.2%) participants in the CBT arm. Of these, 14 (60.9%) attempted the CBT intervention of behavioural activation. Of the nine who did not, four were treatment drop outs, four dealt with mood though general emotional expression which is again classed as counselling and one did not engage with the CBT aspects of treatment.

Anxiety and/or worry was identified as a clinical issue for 20 (54.1%) participants in the CBT arm. Of these, 14 (70%) used a behavioural or cognitive approach to their anxious thoughts. Of the six who did not, three were treatment drop outs, two took a more general counselling approach and one did not engage with CBT. Two further participants identified anger as their main emotional issue; one dropped out and the other was receiving medication and chose not to deal with the anger issues through CBT.

As previously suggested by O’hara [19], perfectionism was also a significant factor in the onset and/or maintenance of dysphonia (i.e. a precipitating or perpetuating factor) in a number of patients in this pilot trial. This was borne out by the treatment formulations. Of the 32 CBT formulations, 17 (53.1%) reported either perfectionism or high self-standards as factors involved in the onset or maintenance of the patient’s problems. Clinically, perfectionism was treated in variety of ways: setting behavioural targets that were not determined by high self-standards; fostering an attitude of self-compassion; cognitive work on “all or nothing” thinking or general counselling skills.

Overall, this content analysis of sessions suggested that CBT treatment was both feasible with, and acceptable to, the majority of participants. By implication, it was also feasible and acceptable to the therapist who was delivering it. This aspect was monitored through case discussion and through listening to audio recordings of CBT sessions and using these as the basis for supervision sessions. Case formulations, treatment targets and treatment rationales were all appropriately employed in all eligible cases. The training took an initial 7 days, and supervision was then weekly for the first year of the trial, becoming every other week and then as needed as the trial progressed and the SLT’s confidence increased. This totalled to approximately 120 h of face to face time between supervisor and therapist over the span of the trial, not including the time taken to prepare and read materials and listen to and reflect on sessions.

#### Clinical utility

For the purposes of this feasibility study, no formal between-group differences are reported. However, what was clear was that at the 6-month follow-up point, there was an effect for both treatment arms (the following figures being for those with complete data at baseline and 6 months). In terms of distress, mean HAD depression scores went from 5.16(sd4.12) to 2.12(sd2.83) in the SLT arm and from 7.08(sd4.5) to 2.69(sd2.83) in CBT arm. Mean GHQ-28 scores went from 29.41(sd14.67) to 14(sd5.61) in the SLT arm and from 30.22(sd15.67) to 16.73(11.04) in the CBT arm. In terms of voice, VPQ scores went from 29.08(sd6.36) to 17.68(sd5.01) in the SLT arm and from 35.28(sd11.42) to 16.77(sd6.53) in the CBT arm. This would suggest that both treatments had a positive effect on voice and distress. However, the utility of the SLT only intervention for distress must be considered in the context of the evidence for CBT co-intervention in the SLT only arm. This is further discussed below. Overall, the use of these candidate outcome measures proved that they are sensitive to change using this intervention for this condition and that they would be suitable for use in a future trial.

## Discussion

This is the first trial to provide feasibility and acceptability data on a cognitive behavioural intervention for functional dysphonia. The data indicates that the transmission of CBT skills to a speech and language therapist was possible and further that the intervention had an impact on the conduct and content of the sessions with patients.

### Feasibility and acceptability

#### As indicated by recruitment and drop out

There was a relatively low refusal rate. Of the 104 who were suitable for randomisation, 30(29%) did not consent to be randomised. This would suggest that the idea of the intervention was acceptable to the majority of patients. Only two gave the content of the intervention as the reason for not wishing to be randomised. The acceptability of the intervention was further attested to by the low drop-out rate by the end of treatment. However, at the 6-month follow-up, there was a higher drop-out of CBT than usual care patients (5 vs 1). Reasons for drop-out were only available for one of the CBT patients (they had moved city). Comparison of drop-outs between conditions showed a trend for participants with higher scores on the VPQ, a measure of general voice-related disability, to drop out of the SLT arm, and participants with lower measures of anxiety, depression and fatigue tended to drop out of the CBT arm. This could be taken to indicate that those who were more disabled by their voice did not feel well served by voice therapy only, whereas those who had relatively less distress did not see the need for the CBT treatment. However, this is only an indicative finding, not definitive. At most it suggests that stratification by vocal disability and general distress might be a part of the design of a future study.

#### As indicated by treatment fidelity and protocol violations

A CBT individualised formulation was possible in 32 out of 37 participants (86.5%). Put another way, in only five cases was there anything like a “pure voice problem”, all the rest had readily elicitable co-morbidities which were amenable to explanation within the CBT model of functional dysphonia. Whilst there was less monitoring of the psychosocial aspects of voice in the usual care, the number of patients where emotional issues became a major part of the work was notable (9 out of 37: 24.3%). It was also clear in these cases that the SLT drew upon CBT techniques and principles to handle these issues. Whilst lacking the formal formulation-based structure of the work done in the CBT arm, the SLT’s existing counselling skills had been substantially augmented by the CBT training, in a way that could not be “undone” when it came to providing usual care. Whilst this co-intervention is a weakness of the trial design, it does indicate that the teaching of CBT skills was feasible and acceptable to the SLT to the extent that they had become internalised and changed routine clinical practice.

From the viewpoint of feasibility, these findings have several implications. First of all, it would seem that emotional issues routinely arise in, and often dominate, voice therapy sessions. Secondly, it is clear that, whilst CBT is a useful framework for addressing these issues, a more general counselling approach, informed by some evidence-based CBT principles, can also be useful, particularly in less-distressed cases. Thirdly, it is clear that even with the best intentions, when faced with distress, it was hard for the SLT not to turn to the newly acquired set of tools. This may explain the impact of both treatments on distress. This is an encouraging finding from the viewpoint of the feasibility of training a speech and language therapist in CBT, but from the viewpoint of a future trial, it would suggest that the influence of CBT training is hard to prevent and certainly needs to be monitored. A design where different therapists deliver the CBT (experimental) and SLT (control) interventions, i.e. a cluster randomised design with treatment allocation at the level of the therapist, would be a preferable design.

#### As indicated by therapy content

The content of the CBT sessions in many ways confirmed the clinical picture of these patients that has been built up in the initial stages of intervention development. This confirmed the utility and acceptability of a CBT model of functional dysphonia to both patients and clinicians. This model was confirmed with regard to not only its form, but also its content. The observation of the importance of fatigue as a co-morbid maintaining factor was borne out. With the exception of voice, fatigue was the major clinical issue in the CBT treatment arm. This was remarkable both as a clinical finding and as a piece of clinical work. It is highly counter-intuitive to seek help for a voice condition and to end up setting targets for daily exercise. If anything could be expected to alienate patients and clinicians, it might be this, and yet it did not. A similar situation occurred with the sleep interventions, the second most common piece of CBT clinical work (67.6% of patients had sleep problems). In the pre-existing skills set of the SLT, there were the means to deal with low mood and worry, but not fatigue or sleep problems. The fact that they were able to identify these symptoms and engage participants in a new treatment for it would indicate that this intervention is highly acceptable to both clinicians and patients. Further, it attests to the SLT’s formulation skills and to the efficacy of CBT formulation in this patient group. Only a good multi-factorial formulation could create a coherent rationale for working on activity and sleep to treat a voice problem. Rationale giving, and a focus on fatigue, should be key points of any future intervention study.

Another finding of this pilot trial was the confirmation of low mood, anxiety and perfectionism in dysphonia. For a future trial, the content of the sessions provides several pointers. The emphasis in training on fatigue, sleep disruption and their treatment was shown to be well founded. Interventions for fatigue and sleep, whilst new to TM, were both readily taught and transmitted to patients, and these should be a central part of a clinical work in general and of any future trial. Behavioural activation for low mood was equally shown to be acceptable and feasible. Cognitive interventions for worry and anxiety perhaps need to be more emphasised in a future training for speech and language therapists delivering CBT, or, given that both groups did equally well on improvements in anxiety, it could be that generic counselling skills are enough for dealing with worry and that these should form part of a future training alongside more specifically CBT techniques. Overall, the CBT treatment content analysis provides strong evidence that CBT was both acceptable to this patient group and this therapist and that its further investigation in a future trial would be feasible.

#### Intervention development

This study also served as a test of the viability of a particular approach to complex intervention development based on the MRC framework [15].This was the first study systemically to develop and pilot a cognitive behavioural intervention for functional dysphonia. The process was one of repeated iteration and evolution, during which the intervention was developed and refined as significant novel clinical findings were added to it. This was in some senses an unexpected by-product of the complex intervention development process. The MRC guidelines for intervention development suggest that the developers build a model of the condition, but not necessarily that this model will expand the general understanding of the condition. However, the sustained and structured scrutiny devoted to functional dysphonia during the intervention development process resulted in both a new model and in the identification of new contributory factors. As Hardeman et al. [13] note, the application of theory to intervention development allows for knowledge of conditions to be cumulative in that theories about mechanism can be tested and refined. To recapitulate this development and evaluation process, a CBT model of medically unexplained symptoms was specified as applicable to the condition and was then piloted as an assessment, formulation and treatment framework in preliminary interviews and through a small consecutive cohort study. This process helped to elaborate the content of the model, to establish that fatigue and perfectionism were clinical issues and to confirm the form of the model, i.e. that it did indeed fit this condition. The data from this first round of evaluation then contributed to both a questionnaire study and a further round of patient interviews which confirmed the utility of the CBT model of MUS as an assessment and formulation structure, and the findings of fatigue and perfectionism as precipitating and perpetuating factors. Based on this, and our increased knowledge of the speech and language profession, the intervention, training, therapist materials, patient materials and outcome measures were re-specified and tested in a pilot RCT of the intervention, in which the acceptability and feasibility to both participants and professionals was examined, through data gathering and through process observation.

### Limitations

One of the chief limitations with this trial is that one therapist delivered both interventions. It was clear from the monitoring of this trial that there was a considerable co-intervention effect. This was ascertained through the clinician’s self-awareness, through the supervisory relationship and through the first author reviewing the recordings of therapy sessions. However, this method of trial monitoring is in itself a limitation, and independent monitoring by an individual not involved in the delivery or supervision of the intervention would be more suitable for a larger scale evaluation. Any future study should employ different therapists to deliver the different treatments. The other major limitation is that patient acceptability was ascertained indirectly through proxies such as drop-out and the form and content of the therapy sessions. A future study should employ patient interviews in addition to these measures.

## Conclusion

This was the development process in essence: a shuttling back and forth between the development and feasibility/piloting stages of the revised MRC framework. As noted above, there is little consensus on how psychosocial interventions should be modelled and piloted. We propose that the methodology outlined above is a valuable systematic process that has allowed not only for a better treatment but also a fuller understanding of the nature of the condition being treated. This work needs to be further investigated in future therapist randomised trials that we hope this work will inform. More specifically, we would reiterate Baker’s [12] insistence on the necessity of training SLTs to assess and treat emotional disorders, and we have shown that a relatively brief training in CBT is a feasible and acceptable way of doing this [12]. These skills are easily generalised to other conditions that SLTs routinely work with. A recently published study by our research group has shown that training a SLT in CBT skills to augment their treatment of swallowing difficulties in head and neck cancer patients was acceptable and feasible to both patients and the clinician [31]. Our current intention is to use the findings from both of these studies to design a CBT training intervention for SLTs and to test its effectiveness in dealing with the psychosocial difficulties associated with a number of issues which commonly present to them, such as dysphonia and swallowing difficulties. Training health professionals in evidence-based psychosocial clinical skills, when conducted systemically and collaboratively, has the potential to significantly impact on patient well-being. The economic evaluation of this approach should be the subject of future studies.

## Abbreviations

CBT: Cognitive behavioural therapy
ENT: Ear Nose and Throat
GHQ: General Health Questionnaire
GRBAS: Grade Roughness Breathiness Asthenia Strain Scale
HAD: Hospital Anxiety And Depression Questionnaire
MRC: Medical Research Council
MUS: Medically unexplained symptoms
SLT: Speech and language therapy/speech and language therapist
VPQ: Vocal Performance Questionnaire

## References

[1] Deary V, Miller T (2011). Reconsidering the role of psychosocial factors in functional dysphonia. Curr Opin Otolaryngol Head Neck Surg.

[2] Ruotsalainen J (2008). Systematic review of the treatment of functional dysphonia and prevention of voice disorders. Otolaryngol Head Neck Surg.

[3] Baker J (2008). The role of psychogenic and psychosocial factors in the development of functional voice disorders. Int J Speech Lang Pathol.

[4] Deary IJ (1997). Personality and psychological distress in dysphonia. Br J Health Psychol.

[5] White A, Deary IJ, Wilson JA (1997). Psychiatric disturbance and personality traits in dysphonic patients. Eur J Disord Commun.

[6] MacKenzie K (2001). Is voice therapy an effective treatment for dysphonia? A randomised controlled trial. Br Med J.

[7] Nimnuan C, Hotopf M, Wessely S (2001). Medically unexplained symptoms—an epidemiological study in seven specialities. J Psychosom Res.

[8] olde Hartman TC, et al. What do guidelines and systematic reviews tell us about the management of medically unexplained symptoms in primary care? BJGP Open. 2017: p. BJGP-2016-0868. 10.3399/bjgpopen17X101061 PMC6169926 30564678

[9] Deary V, Chalder T, Sharpe M (2007). The cognitive behavioural model of medically unexplained symptoms: a theoretical and empirical review. Clin Psychol Rev.

[10] Kennedy TM (2006). Cognitive behavioural therapy in addition to antispasmodic therapy for irritable bowel syndrome in primary care: randomised controlled trial. Health Technol Assess.

[11] Ismail K (2008). Motivational enhancement therapy with and without cognitive behavior therapy to treat type 1 diabetes. Ann Intern Med.

[12] Baker J (2010). Women’s voices: lost or mislaid, stolen or strayed?. Int J Speech Lang Pathol.

[13] Hardeman W (2005). A causal modelling approach to the development of theory-based behaviour change programmes for trial evaluation. Health Educ Res.

[14] Lovell K (2008). Developing guided self-help for depression using the Medical Research Council complex interventions framework: a description of the modelling phase and results of an exploratory randomised controlled trial. BMC Psychiatry.

[15] Craig P (2008). Developing and evaluating complex interventions: the new Medical Research Council guidance. BMJ.

[16] Michie S (2008). Behavior matters: what works in self-regulation interventions?. Int J Psychol.

[17] Clark DM (2004). Developing new treatments: on the interplay between theories, experimental science and clinical innovation. Behav Res Ther.

[18] Daniilidou P (2007). Cognitive behavioral therapy for functional dysphonia: a pilot study. Ann Otol Rhinol Laryngol.

[19] O’Hara J (2011). Relationship between fatigue, perfectionism, and functional dysphonia. Otolaryngol Head Neck Surg.

[20] Hirano M (1981). Clinical examination of voice.

[21] Carding PN, Horsley IA, Docherty GJ (1999). A study of the effectiveness of voice therapy in the treatment of 45 patients with nonorganic dysphonia. J Voice.

[22] Cuijpers P, van Straten A, Warmerdam L (2007). Behavioral activation treatments of depression: a meta-analysis. Clin Psychol Rev.

[23] Abramowitz, J.S., B.J. Deacon, and S.P. Whiteside, Exposure therapy for anxiety: principles and practice. UK: Guilford Press; 2011.

[24] Deary V, Chalder T (2010). Personality and perfectionism in chronic fatigue syndrome: a closer look. Psychol Health.

[25] Lancaster GA, Dodd S, Williamson PR (2004). Design and analysis of pilot studies: recommendations for good practice. J Eval Clin Pract.

[26] Deary V. Cognitive behavioural therapy for functional dysphonia: development of a complex intervetion. PhD thesis, 2011. Institute of Health and Society, Newcastle University, UK.

[27] Eldridge SM (2016). CONSORT 2010 statement: extension to randomised pilot and feasibility trials. Pilot Feasibility Stud.

[28] Golderberg D, Williams P. A user's guide to the General Health Questionnaire. Windsor, UK: NFER-Nelson. 1988.

[29] Zigmond AS, Snaith RP (1983). The hospital anxiety and depression scale. Acta Psychiatr Scand.

[30] Pawlikowska T (1994). Population-based study of fatigue and psychological distress. Br Med J.

[31] Patterson J (2018). Feasibility and acceptability of combining cognitive behavioural therapy techniques with swallowing therapy in head and neck cancer dysphagia. BMC Cancer.

